# Eleutheroside K Isolated from *Acanthopanax henryi* (Oliv.) Harms Inhibits the Expression of Virulence-Related Exoproteins in Methicillin-Resistant *Staphylococcus aureus*

**DOI:** 10.1007/s00284-021-02631-5

**Published:** 2021-09-23

**Authors:** Qian-Qian Li, Jiao Luo, Xiang-Qian Liu, Ok-Hwa Kang, Dong-Yeul Kwon

**Affiliations:** 1 Department of Oriental Pharmacy, College of Pharmacy and Wonkwang Oriental Medicines Research Institute, Wonkwang University, Iksan, Jeonbuk 54538 Republic of Korea; 2 School of Pharmacy, Hunan University of Chinese Medicine, Changsha, Hunan 410-208 People’s Republic of China

## Abstract

Methicillin-resistant *Staphylococcus* (*S.*) *aureus* (MRSA) is a representative pathogen that produces numerous virulence factors involving manifold cytotoxins and exotoxins. The present study was designed to investigate the influence of Eleutheroside K (ETSK), a single compound isolated from the leaves of *Acanthopanax* (*A.*) *henryi* (Oliv.) Harms, on the exotoxins secreted by MRSA. The transcription and translation of the exotoxins (α-hemolysin and staphylococcal enterotoxins) related to virulence in *S. aureus* were determined via quantitative RT-PCR and western blot analysis. The effect of ETSK on the production of tumor necrosis factor (TNF)-α was evaluated using enzyme-linked immunosorbent assay. As a result, ETSK at sub-MIC concentrations could reduce the protein expression of α-hemolysin and enterotoxin, and the expression of genes that regulate virulence factors was also inhibited. In addition, the TNF-inducing activity of *S. aureus* was attenuated by ETSK in a dose-dependent manner. These results revealed that ETSK not only reduced the protein and gene expression levels of related exotoxins but also suppressed the ability of *S. aureus* to induce macrophages to release cytokines. This study indicated that the inhibition of MRSA infection by ETSK may be achieved by reducing the virulence of *S. aureus* and highlighted the potential of ETSK as an innovative strategy for the prevention and treatment of MRSA infections.

**Supplementary Information:**

The online version contains supplementary material available at 10.1007/s00284-021-02631-5.

## Introduction

*Staphylococcus* (*S.*) *aureus* is a causal organism implicated in considerable opportunistic infections, colonizing humans and animals [1]. It is a substantial contributor to a variety of invasive infectious diseases and can penetrate the subcutaneous tissues to reach the blood [2]. In order to inhibit bacterial growth, numerous antibiotics have been developed and used to treat *S. aureus* infections [3]. However, the efficacy of antibiotics was limited by the emergence of methicillin-resistant *S. aureus* (MRSA) [4, 5]. Since MRSA is recognized as one of the major causes of healthcare- and community-associated infections and is a serious menace that frequently occurs all over the world, the treatment of *S. aureus* infections becomes complicated [6, 7]. The community is facing a huge challenge, and MRSA infection is especially concerning. The pathogenicity of MRSA is predominantly attributed to the toxin production and drug resistance. Exploring drugs that can interfere with the synthesis of bacterial virulence factors may be a countermeasure against MRSA infection [8].

Several *S. aureus* exotoxins produced by MRSA can exacerbate the symptoms of MRSA infections and play a crucial role in the pathogenesis, including hemolysins, enterotoxins, toxic shock syndrome toxin 1 (TSST-1) and panton–valentine leukocidin (PVL) [9]. These toxins are related to the increased inflammation and disease severity of *S. aureus* infection. In this study, we mainly focused on the exotoxin genes and virulence proteins, including α-hemolysin and staphylococcal enterotoxins (SEs) produced by MRSA. The expression of multiple virulence factors produced by *S. aureus* is strictly regulated. The global regulatory systems such as accessory gene regulators (*agr*) system control the expression of *S. aureus* toxins [10]. The *agr* locus is considered to be the quorum-sensing system of *S. aureus*, and RNAII is the operon of *agr*BDCA driven by promoter 2 (P2). AgrB is encoded as a transmembrane endopeptidase and is responsible for the processing and export of AgrD, which is the precursor of autoinducer peptide (AIP). At a threshold concentration, AIP binds to AgrC to autophosphorylate AgrC and subsequently phosphorylate AgrA. The activated AgrA triggers P3-driven transcription followed by translation of RNAIII [11]. RNAIII is the main effector of *agr* system, which can positively regulate the expression of α-hemolysin and SEs [12].

Previous studies have reported that bacterial virulence factors mediate *S. aureus*-associated infections [13]. Multiple proteins such as α-hemolysin and enterotoxins are predominant virulence factors secreted by *S. aureus* strains. The α-hemolysin produced by most pathogenic strains of *S. aureus* is a 33-kDa pore-forming toxin that exhibits dermonecrotic, cytolytic, hemolytic, and lethal activities [14]. The SEs secreted by certain *S. aureus* strains can induce staphylococcal gastroenteritis and lead to food poisoning in humans [15]. At the transcriptional level, α-hemolysin, SEA, and SEB are coded by *hla*, *sea*, and *seb*, respectively [16]. Moreover, the *agr* are recognized as a distinct global regulatory system that encodes the gene expression of manifold virulence factors [17]. In addition, SEs stimulate T-cell activation to release T cell-derived cytokines [14]. Cytokines such as interleukin-6 and tumor necrosis factor (TNF)-α exhibit pro-inflammatory effects in multiple inflammatory disorders [18]. It has been reported that MRSA has the potential to up-regulate the pro-inflammatory levels of TNF-α secreted by macrophages [19].

To address the intractable infections, the unique research idea of studying the antibacterial effects of naturally occurring compounds may lead to a novel treatment strategy. *Acanthopanax* (*A.*) *henryi* (Oliv.) Harms belongs to family *Araliaceae* and is widely distributed in China, Korea, and Japan. *A. henryi* (Oliv.) Harms has been used extensively as a traditional oriental medicine to treat rheumatism and cancer [20]. According to reports, the root bark of *A. henryi* has significant anti-inflammatory activity, while the leaves of *A. henryi* possess anti-oxidant activity [21, 22]. Various bioactive ingredients have been extracted from *A. henryi* (Oliv.) Harms [23]. The present study focused on Eleutheroside K (ETSK) (Suppl. Fig. 1), one of the bioactive compounds extracted from the leaves of *A. henryi*, as a latent therapeutic agent to eliminate bacterial virulence factors, so as to achieve the purpose of inhibiting MRSA infection. There are few studies on the pharmacological activity of ETSK. By determining the antibacterial susceptibility, our previous studies confirmed the pharmacological mechanism of ETSK reversing the methicillin resistance caused by MRSA and displayed a minimal inhibitory concentration of 50 µg/mL [24]. In this context, the antibacterial ability of ETSK will be further investigated by analyzing the effect of ETSK on the secretion of staphylococcal virulence factors in vitro.

## Materials and Methods

### Plant Collection

The leaves of *A. henryi* (Oliv.) Harms were collected in October 2012 at Xinhua (Changsha, China). The plant species was confirmed by Professor Xiang-Qian Liu (Hunan Key Laboratory of Traditional Chinese Medicine Modernization, Hunan University of Chinese Medicine, Changsha, China) and the voucher specimen (No. 20121125) was deposited at the School of Pharmacy, Hunan University of Chinese Medicine.

### Extraction and Isolation

The dried leaves of *A. henryi* (Oliv.) Harms (10 kg) were extracted with methanol and the resulting extract was partitioned between petroleum ether and H_2_O. The fractionation of water fraction was done using column chromatography (CC) on macroporous resin. Finally, the water fraction was eluted into five fractions under a gradient of EtOH/H_2_O (0, 30, 50, 75, and 95%). Fraction 4 (75% EtOH, 14.0 g) was subjected to various CC [silica gel, Sephadex LH-20, and reverse-phase octadecylsilyl (ODS) preparative high-performance liquid chromatography (HPLC)] to obtain compound ETSK [25]. The structure of ETSK was identified using mass spectroscopy, 1D nuclear magnetic resonance (NMR), and 2D NMR. As previously described, the purity of ETSK determined by HPLC was > 98% [18].

### Reagents

Difco™ Mueller–Hinton agar (MHA), Difco™ Mueller–Hinton broth (MHB), and Difco™ skim milk were obtained from Difco Laboratories (Baltimore, MD, USA). Glycerol was obtained from Sigma-Aldrich; Merck KGaA (Darmstadt, Germany). Power SYBR® Green PCR master mix was purchased from Applied Biosystems (Warrington, UK). SMART™ bacterial protein extraction solution was purchased from Intron Biotechnology Inc. (Seongnam, Korea). Mouse TNF (Mono/Mono) ELISA set and the 3, 3′, 5, 5′-tetramethyl benzidine (TMB) Substrate Reagent Set was purchased from BD OptEIA™ (BD Biosciences, San Diego, CA92121, USA).

### Bacterial Strains and Culture Medium

MRSA strain ATCC 33,591 was purchased from the American Type Culture Collection (Manassas, VA, USA). ATCC 33,591 was cultured on either MHA or MHB at 37 °C for 24 h for experimental use. The bacteria were stored in 30% glycerol and frozen at − 80 °C.

### RNA Extraction and Quantitative Real-Time Reverse Transcription Polymerase Chain Reaction (qRT-PCR)

Real-time RT-PCR was performed as previously described [26]. In our previous experiment, it has been determined that the minimum inhibitory concentration of ETSK against ATCC 33,591 is 50 µg/mL [24]. The ATCC 33,591 strain was grown to an absorbance value of 0.9 at an optical density 600 nm (OD_600nm_) and treated with sub-inhibitory concentrations (6.25–25 µg/mL) of ETSK for 4 h [26]. Bacterial cells were pelleted by centrifugation at 13,000 g for 10 min. Total RNA was prepared using the E.Z.N.A.® bacterial RNA kit (OMEGA Bio-Tek, Norcross GA, USA) according to the manufacturer’s protocol. The mRNA concentrations were measured using Nanodrop spectrophotometer (Bio-Tek, Winooski, VT, USA), and equal mRNA amounts (1 μg) were adjusted using gDNA Wipeout Buffer and RNase-free water according to the calculated mRNA concentrations. The QuantiTect reverse transcription kit (Qiagen, Seoul, Korea) was used to reverse transcribe mRNA into the first-strand complementary DNA (cDNA), and 14 μL sample mRNA, 1 μL reverse transcriptase (RT), 1 μL gene-specific primers (0.7 μM), and 4 μL RT buffer were mixed to obtain sample cDNA. Components needed to run PCR were set up as follows: 2 μL sample cDNA, 10 μL SYBR master mix, 1 μL of each primer (10 μM), and 6 μL deionized water to a total volume of 20 μL. The primer pairs used to synthesize the DNA template for qRT-PCR are presented in Suppl. Table 1. The quantitative PCR was run using the StepOnePlus real-time PCR system (Applied Biosystems, France).

### Protein Extraction and Western Blotting Analysis

The western blot was performed as described previously to measure protein translation levels [26]. ATCC 33,591 suspensions (OD_600nm_ value of 0.9) were treated with graded sub-inhibitory concentrations (6.25–25 µg/mL) of ETSK. Bacterial cells were harvested after 4 h [26] and suspended in bacterial protein extraction solution according to the manufacturer’s instructions. Bacterial lysates were centrifuged at 13,000× *g* for 10 min to remove the insoluble fractions. Equal protein amounts were measured using Bio-Rad protein assay reagent (Bio-Rad Laboratories, Inc., Hercules, CA, USA) to perform the sodium dodecyl sulfate–polyacrylamide gel electrophoresis (SDS-PAGE). The electrophoresed gels were transferred to Amersham™ Hybond™-P-membranes (GE Healthcare, Piscataway, NJ, USA). The membranes were blocked in Tris-buffered saline with Tween 20 (TBST) containing 5% skim milk and then probed with polyclonal rabbit anti-Staphylococcus alpha-hemolysin antibody (Abcam, UK), polyclonal rabbit anti-Staphylococcus Enterotoxin A antibody ab 15,897 (Abcam, UK), and polyclonal rabbit anti-Staphylococcus Enterotoxin B antibody ab 15,898 (Abcam, UK). The loading differences were normalized with mouse monoclonal anti-glyceraldehyde 3-phosphate dehydrogenase (GAPDH) antibody (Cell signaling technology, USA). The membranes were incubated overnight at 4 °C. After washing three times, membranes were re-probed with goat anti-rabbit IgG secondary antibody (Thermo Scientific, USA) and anti-mouse IgG secondary antibody (Enzo Life Sciences, USA) for 2 h. The membranes were then supplemented with TOPview™ ECL Femto Western Substrate (Enzynomics, Korea). Imagequant LAS-4000 mini chemical luminescent imager (GE Healthcare Life, Korea) was used to detect the immunoreactive bands of membranes.

### Enzyme-Linked Immunosorbent Assay (ELISA)

ATCC 33,591 (OD_600nm_ value of 0.3) was grown by shaking overnight in MHB with graded concentrations of ETSK (1/8, 1/4, and 1/2 × MIC). Untreated bacteria were used as a control. After centrifuging at 4000 g for 10 min, the collected supernatants (protein secretions) were filtered through 0.45 μM micro filters. While, RAW 264.7 cells were seeded at a density of 10^6^/mL in RPMI 1640 (supplemented with 10% FBS, 100 IU/mL penicillin, and streptomycin). The cell suspension (100 μL) was dispensed into a 96-well cell culture plate and then incubated at 37 °C in an incubator (5% CO_2_) for 18 h to make cells adhere. When cells adhered to the bottom of the plate, cell culture media were replaced with fresh RPMI 1640 medium (150 μL). The filtered *S. aureus* supernatants (50 μL) were added to the corresponding wells. Fresh RPMI was used instead of bacterial supernatant as normal blank control group. After incubation for 24 h, the culture medium of RAW 264.7 cells was collected in 1.5 mL tubes and centrifuged (13,000 *g* for 5 min) to obtain the supernatant samples.

The tumor necrosis factor (TNF) level was measured using the OptEIA™ mouse TNF (Mono/Mono) ELISA set according to the manufacturer’s instructions. The required wells of 96-well plate were coated with the capture antibody diluted in coating buffer (1:250), then the plate was sealed, and incubated overnight at 4 °C. After washing each well with phosphate-buffered saline (PBS) (0.05% Tween 20) three times, the non-specific binding sites were blocked with assay buffer (PBS-containing 10% FBS) for 1 h. After washing 3 times, 100 μL sample and TNF-α standard were added to the corresponding wells and incubated at room temperature for 2 h. After washing 5 times, 100 μL detection antibody and Enzyme Reagent SAv-HRP diluted in assay buffer (1:250, respectively) were added to each well and incubated for 1 h. After washing the wells 7 times, 100 μL TMB substrate solution (1:1) was added to each well and incubated at room temperature in the dark. When a clear blue appeared, 50 μL stop solution (2 M H_2_SO_4_) was added to each well. The final liquid was transferred in each well to a clean new 96-well plate, and the absorbance values were measured at an OD of 450 nm using spectrophotometer.

### Statistical Analysis

All experiments were performed in triplicate and the data are presented as the mean ± standard deviation (SD). IBM SPSS statistics 24 was performed using one-way analysis of variance (ANOVA) and Duncan’s multiple range test (DMRT) to analyze statistical differences between the mean values (*P* value < 0.05).

## Results

### ETSK Represses the Transcription of *hla*, *sea*, *seb*, and *agrA* Genes in *S. aureus*

**Fig. 1:**
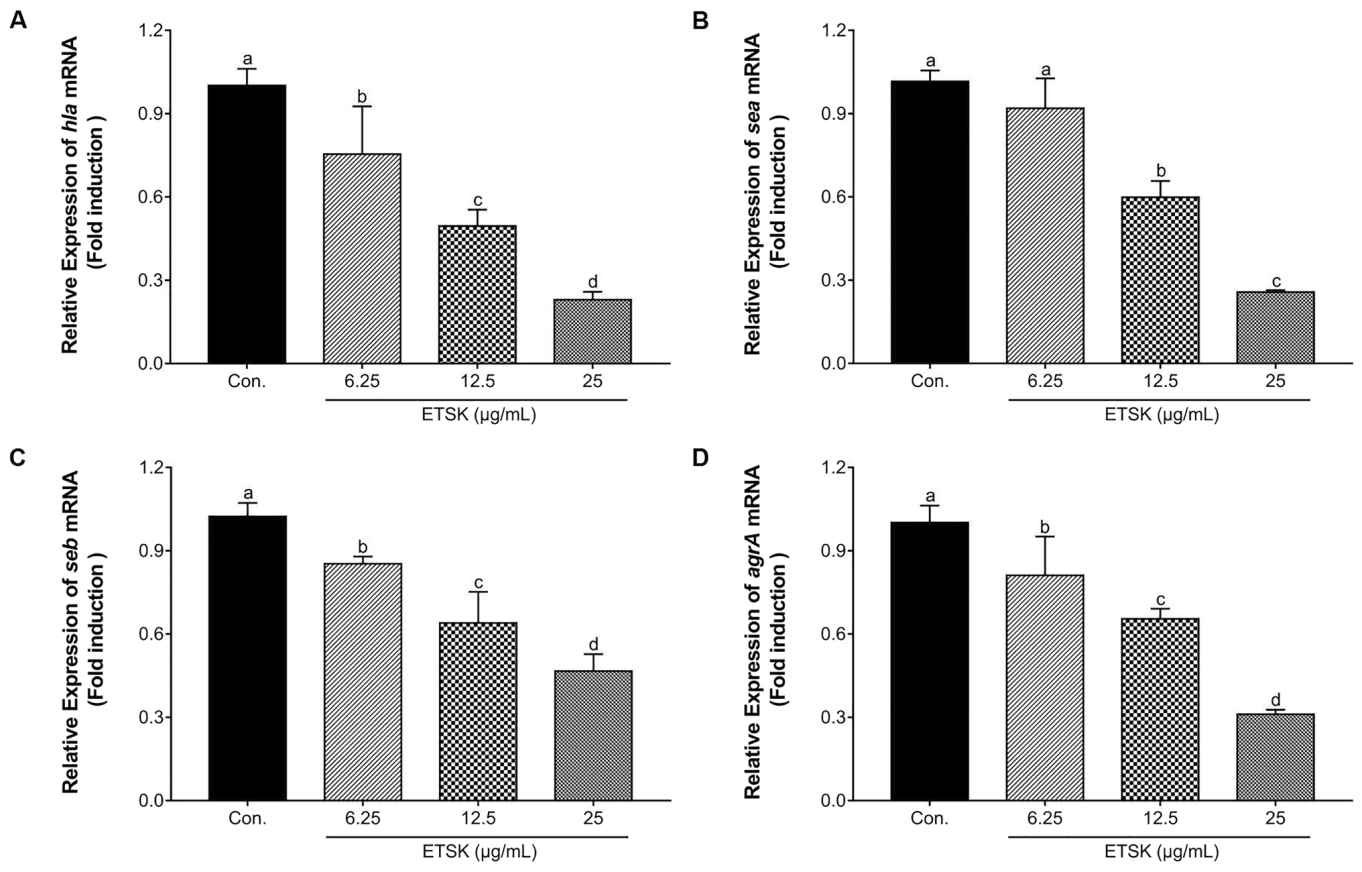
Relative gene expression of *hla* (**A**), *sea* (**B**), *seb* (**C**), and *agrA* (**D**) in *S. aureus* ATCC 33,591 grown under graded sub-inhibitory concentrations (6.25–25 µg/mL) of ETSK was analyzed by quantitative RT-PCR (qRT-PCR). Values represent the means ± SD of three independent experiments. Different letters indicate statistically significant differences (*P* < 0.05), while the same letters mean no significant differences (*P* > 0.05). Con., control, untreated *S. aureus* strains; *hla*, gene encoding α-hemolysin; *sea*, gene encoding staphylococcal enterotoxin A; *seb*, gene encoding staphylococcal enterotoxin B; *agrA*, gene encoding accessory gene regulator A; ETSK, Eleutheroside K

Quantitative reverse transcription PCR (qRT-PCR) was performed to investigate the effect of ETSK on the expression of genes encoding α-hemolysin, SEA, and SEB. The *agrA* is a critical operon that regulates the transcription of these toxic factor genes. As shown in Fig. 1, upon treatment with ETSK at sub-MIC concentrations, the expression of *sea*, *seb*, *hla*, and *agrA* in ATCC 33,591 were significantly reduced in a dose-dependent manner. In the treatment group, the downward trend in gene expression of toxicity-related factors suggests that ETSK may prevent MRSA infection by inhibiting representative exotoxin produced by MRSA. Therefore, the next experiment aims to determine whether the protein translation process of these exotoxin is affected by ETSK as expected.

### ETSK Represses the Translation of α-hemolysin and Staphylococcal Enterotoxins in *S. aureus*

**Fig. 2:**
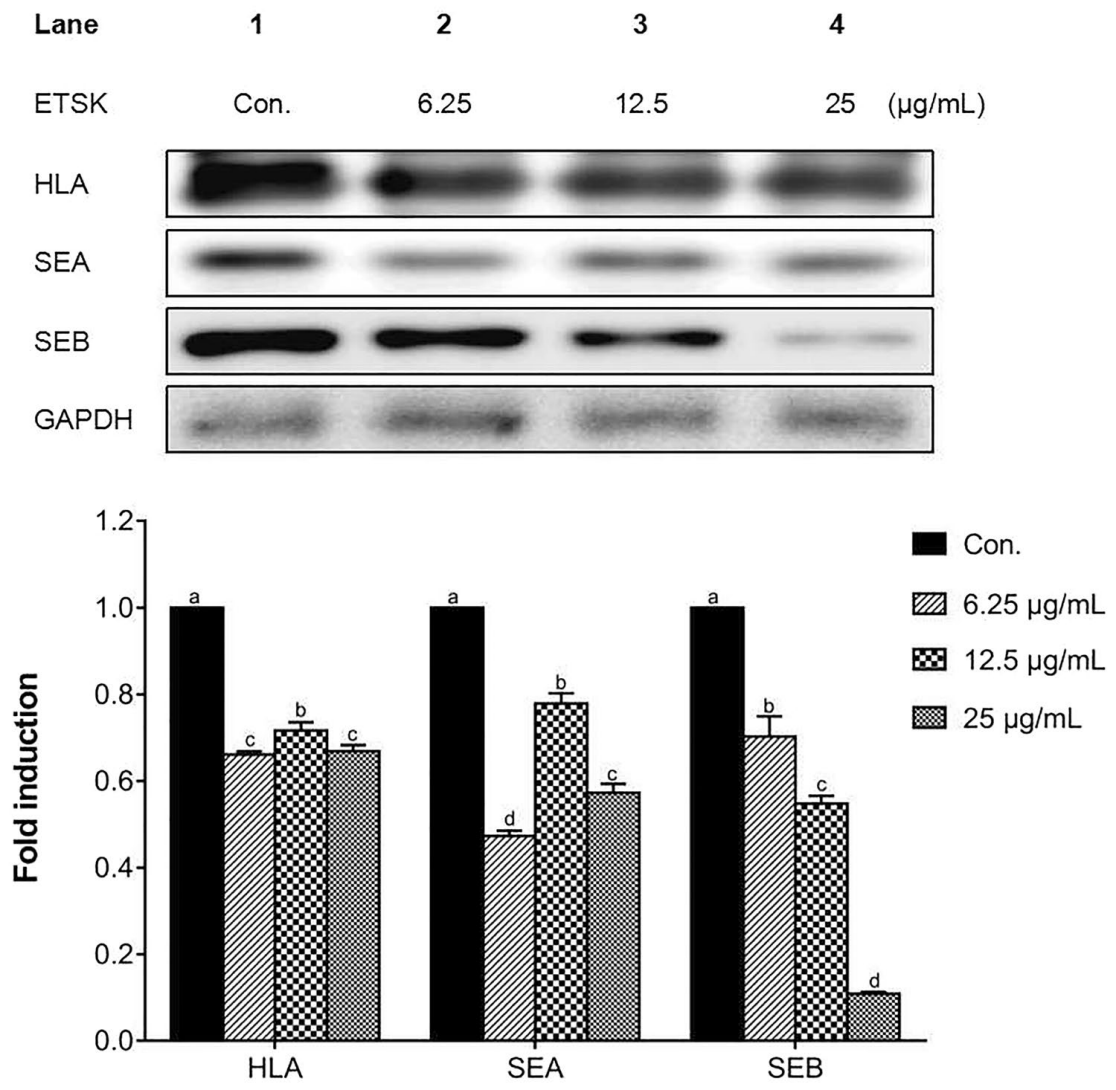
The protein expression of α-hemolysin, SEA, and SEB produced by ATCC 33,591 strain treated with the sub-inhibitory concentrations (6.25–25 µg/mL) of ETSK were analyzed by western blot. After 4 h of treatment, ETSK with graded sub-inhibitory concentrations suppressed the production of α-hemolysin, SEA, and SEB. GAPDH was used as the loading control. Values represent the means ± SD of three independent experiments. Different letters indicate statistically significant differences (*P* < 0.05), while the same letters mean no significant differences (*P* > 0.05). Con., control, untreated *S. aureus* strain; HLA, α-hemolysin; SEA, staphylococcal enterotoxin A; SEB, staphylococcal enterotoxin B; GAPDH, glyceraldehyde 3-phosphate dehydrogenase; ETSK, Eleutheroside K

The secretion of α-hemolysin and staphylococcal enterotoxins by ATCC 33,591 exposed to sub-inhibitory concentrations of ETSK was determined by western blotting analysis. Figure 2 illustrates the expression of these three representative *S. aureus* exotoxins after treatment with ETSK for 4 h. In the presence of sub-inhibitory concentrations of ESTK, although the dose-dependent manner is not obvious, it can be seen that the secretion of α-hemolysin and SEA was significantly reduced compared with the untreated group. According to the western blot analysis of SEB, the inhibitory effect of ETSK on SEB is obviously dose-dependent. The original and un-cropped images of the results were presented in Suppl. Figure 2. These results reinforce the notion that the mechanism by which ETSK inhibits MRSA infection is related to the attenuation of *S. aureus* exotoxins expression by ETSK.

### ETSK Represses TNF-Inducing Activity of *S. aureus*

**Fig. 3:**
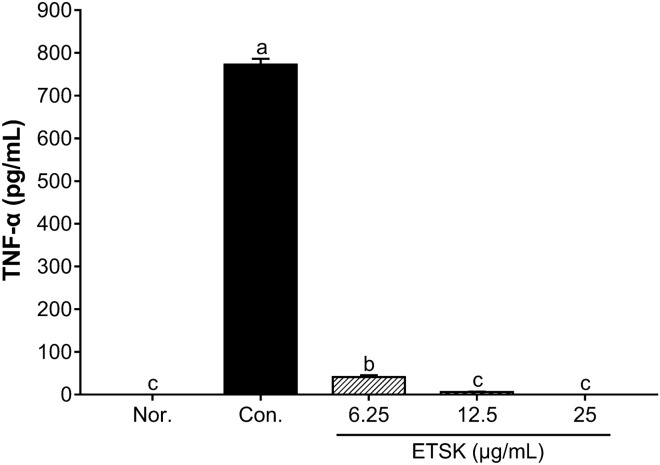
Enzyme-linked immunosorbent assay (ELISA) analysis of tumor necrosis factor (TNF) released by RAW 264.7 cells stimulated with the supernatants of *S. aureus* grown in the presence of graded sub-inhibitory concentrations (6.25–25 µg/mL) of ETSK. Values represent the means ± SD of three independent experiments. Different letters indicate statistically significant differences (*P* < 0.05), while the same letters mean no significant differences (*P* > 0.05). Nor., normal, blank control group; Con., control, untreated *S. aureus* strain; ETSK, Eleutheroside K

Enzyme-linked immunosorbent assay (ELISA) was performed to evaluate the effect of ETSK on the production of pro-inflammatory cytokine TNF-α. *S. aureus* infection can cause a series of inflammatory disorders. It has been reported that MRSA has the ability to induce the release of pro-inflammatory cytokines from T cells [15]. SEs are important exotoxins produced by *S. aureus* and can act as superantigens to stimulate macrophages to produce cytokines. In order to verify the effect of ETSK on the inflammation of macrophage RAW 264.7 induced by *S. aureus* exotoxins, we tested the expression of the representative pro-inflammatory cytokine TNF-α. Figure 3 depicts that the presence of MRSA culture supernatant triggers the massive release of TNF-α from RAW 264.7 compared with the normal blank control group. However, the culture supernatants of ATCC 33,591 grown under sub-inhibitory concentrations of ETSK apparently hampered TNF-inducing activity of *S. aureus* in a dose-dependent manner.

## Discussion

*S. aureus* is a ubiquitous pathogen that can cause a wide spectrum of infectious diseases, including skin and soft tissue infections [27]. It is one of the main causes of bacterial infections worldwide [28]. Since the emergence of MRSA in 1961, there have been serious infection control problems [29]. The pathogenesis of MRSA is diverse. Manifold antibiotics mainly aimed at inhibiting the growth of *S. aureus* that have been developed and applied to treat *S. aureus* infections. However, due to the growing emergence of multi-drug-resistant bacteria, the development of effective antibacterial drugs or novel therapeutic approaches has become more urgent [30]. Additionally, the virulence factors produced by *S. aureus* enable the bacterial pathogens to engender various troublesome infections. These virulence factors (such as exotoxins and enzymes) can be superantigenic and can transform host tissues into nutrients needed for bacterial growth to help *S. aureus* pathogens survive under various conditions [26]. Also, many *S. aureus* can express adhesion molecules to make bacteria adhere to host cells, which promotes the formation of biofilms, making *S. aureus* infections increasingly difficult to treat [31]. In addition to inhibiting the essential processes of bacterial growth and survival, an innovative exploratory approach for ameliorating the condition is to find novel agents that target bacterial virulence factors. Studies related to the antibacterial activity and mechanism of plant extracts have been reported previously [32]. ETSK (oleanolic acid 3–O–α-l-rhamnopyranosyl-(1 → 2)-α-l-arabinopyranoside), as an active oleanane-type triterpenoid saponin isolated from the leaves of the renowned medicinal plant *A. henryi* (Oliv.) Harms, has attracted our attention to study whether it has the ability to treat MRSA infections. Since ETSK bears a unique disaccharide moiety that induces cytotoxicity, it generally has significant anti-tumor activity [33]. Our earlier research on the effect of natural pharmaceutical compound ETSK on reversing the drug resistance caused by MRSA aroused our curiosity about the potential of ETSK in inhibiting the expression of related virulence factors. In the present study, we explored the medicinal mechanism of ETSK by investigating the inhibitory effect of ETSK on the synthesis of *S. aureus* exotoxins.

The α-hemolysin is a crucial virulence factor secreted by the majority of *S. aureus* strains and can subvert the host immune system. As a cell membrane pore-forming toxin, α-hemolysin is primarily responsible for the hemolytic activity of *S. aureus* and can engender tissue damage. In addition, in epithelial cells, the escape of *S. aureus* from endocytic vesicles into the cytosol requires α-hemolysin [34]. The α-hemolysin is widely known to induce cytotoxicity against a variety of cells and plays a predominant role in the pathogenesis of severe cases of pneumonia [35] Based on analysis of gene and protein level expression, in the untreated control group, the high expression of α-hemolysin is very obvious. Compared with the control group, the protein and gene expression of α-hemolysin were obviously diminished in the presence of sub-inhibitory concentrations of ETSK. However, the trend between transcription level and translation level is not completely consistent, which may be caused by the modification, transport, and degradation of mRNA during protein translation. In general, it can be concluded that the mechanism by which ETSK inhibits MRSA infection is probably attributed to the blockade of the main toxin product α-hemolysin.

SEs have been recognized as bacterial superantigens that induce cellular proliferation, cytokine storm, and toxic shock syndrome [36]. Additionally, the emetic activity of SEs and the resistance to inactivation of SEs caused by gastrointestinal proteases have potential to trigger staphylococcal gastroenteritis and food poisoning in humans [34]. Due to these characteristics of SEs, research to effectively inhibit these exotoxin has become an urgent demand. Five major types based on serology (SEA to SEE) have been classified. Meanwhile, more than 20 distinct types of SEs with consensus sequence homology are known; however, only a few of them have been studied in depth. SEA and SEB are the most common SEs. SEA is the most usual cause that prompts staphylococcus-related food-borne poisoning, while SEB is also related to food poisoning and is assumed to be applied as an inhaled biological weapon [37]. According to our qRT-PCR and western blotting analysis results, ETSK has an inhibitory effect on the SEs gene and protein expression at sub-inhibitory concentrations. The salient inhibitory effect of ETSK on gene transcription and protein translation of MRSA virulence factors (such as α-hemolysin and SEs) at sub-inhibitory concentrations indicates the potential of ETSK to be applied to the treatment of MRSA infections. The gene and protein expression of SEB showed consistent dose-dependent reduction. However, although the gene transcription and the protein translation of SEA were suppressed by ETSK, there is not a completely consistent weakening trend. As for the reason for this phenomenon, we speculate that, in addition to the possibility of related mRNA modification and degradation, there may also be some small mRNA regulation effects, such as miRNA, during protein translation. In addition, our research also attempted to explore the effect of ETSK on the *agr* system. In the presence of ETSK, the *agrA* was inhibited at the gene level in a dose-dependent manner. We speculate that ETSK suppresses the expression of genes encoding *S. aureus* exotoxin downstream of *agrA* by inhibiting the gene expression of the *agr* regulatory system. Due to the down-regulation of *agrA* gene expression, we have sufficient reasons to infer that the expression of *S. aureus* exotoxins regulated by RNAIII will be suppressed at both gene and protein levels [38, 39]. As a factor that activates RNAIII expression, the downward trend of *agrA* gene expression is consistent with the inhibition of α-hemolysin and SEs as expected.

The SEs are the most important exotoxins of *S. aureus* that act as superantigens, which could induce the release of pro-inflammatory cytokines by T cells [14]. Therefore, to elucidate the biological relevance of reduced staphylococcal exotoxin secretion caused by ETSK, Enzyme-linked immunosorbent assay (ELISA) was performed to evaluate the effect of ETSK on the production of pro-inflammatory cytokine TNF-α. The ability of ETSK to down-regulate TNF-α was observed in our present study. Compared to the untreated normal group, the plethora of TNF-α release is caused by the supernatant of *S. aureus* culture medium, while in the presence of culture supernatants of *S. aureus* grown under sub-inhibitory concentrations of ETSK, the content of TNF-α was substantially reduced dose dependently. This reduction of TNF-α released by RAW 264.7 suggests the potential of ETSK to treat the inflammation caused by MRSA infection. In addition to inhibiting the gene and protein expression of *S. aureus* exotoxins, we believe that ETSK also shows excellent anti-inflammatory ability [40]. From the molecular level, we speculate that the therapeutic effect of ETSK on inflammation is related to the inhibition of SEs by ETSK treatment. Since cytokines are released under the stimulation of SEs, the reduction of SEs can be inferred from the result of the diminished TNF-inducing ability. Based on the determination of the protein and gene expression of SEs, our conjecture is considered reliable.

According to the foregoing findings, the authors infer that ETSK may inhibit the production of exotoxin by *S. aureus* not only during protein translation but also at the level of gene transcription. ETSK significantly down-regulated the expression of virulence-related factors of *S. aureus* and the higher the concentration of ETSK, the greater its usefulness to impede the toxin production. In addition, this study also found that at a higher bacterial density (OD_600nm_ = 0.9), ETSK still showed a significant inhibitory effect on toxins produced by MRSA, even if the strains were subjected to less drug pressure. Overall, the present results suggest the potential of ETSK as a novel antibacterial compound for the prevention and control of serious and urgent MRSA infection.

## Conclusion

This study was devoted to investigating the capability of ETSK isolated from the leaves of *A. henryi* (Oliv.) Harms to repress the virulence factors released by *S. aureus*. The results indicated that sub-inhibitory concentrations of ETSK impeded the protein and gene expression of *S. aureus* exotoxins, and it was even possible to reduce the ability of *S. aureus* to induce macrophages to release pro-inflammatory cytokines by inhibiting the exotoxin SEs. The hypothesis that ETSK has an inhibitory effect on the virulence-related exoproteins of MRSA has been confirmed. Therefore, we believe that ETSK can be used as an innovative antibacterial drug to ameliorate the intractable MRSA infection by hindering the production of exotoxins. Herein, we propose the concept that the natural plant compound ETSK possesses the ability to inhibit the toxicity of MRSA. In order for ETSK to be truly applied to the clinical treatment of difficult MRSA infections, more in-depth exploration and research on ETSK are needed.

## Supplementary Information

Below is the link to the electronic supplementary material.

Supplementary file1 (DOCX 76 kb)

Supplementary file2 (DOCX 1375 kb)

Supplementary file3 (DOCX 12 kb)

## Data Availability

The data used or analyzed during the present study are available from the corresponding author on reasonable request.
