# Supplementary figures and images for: Eleutheroside K Isolated from Acanthopanax henryi (Oliv.) Harms Inhibits the Expression of Virulence-Related Exoproteins in Methicillin-Resistant Staphylococcus aureus
Source: Curr Microbiol. 2021 Sep 23;78(11):3980–8. doi: 10.1007/s00284-021-02631-5 (PMC8486718; doi:10.1007/s00284-021-02631-5)

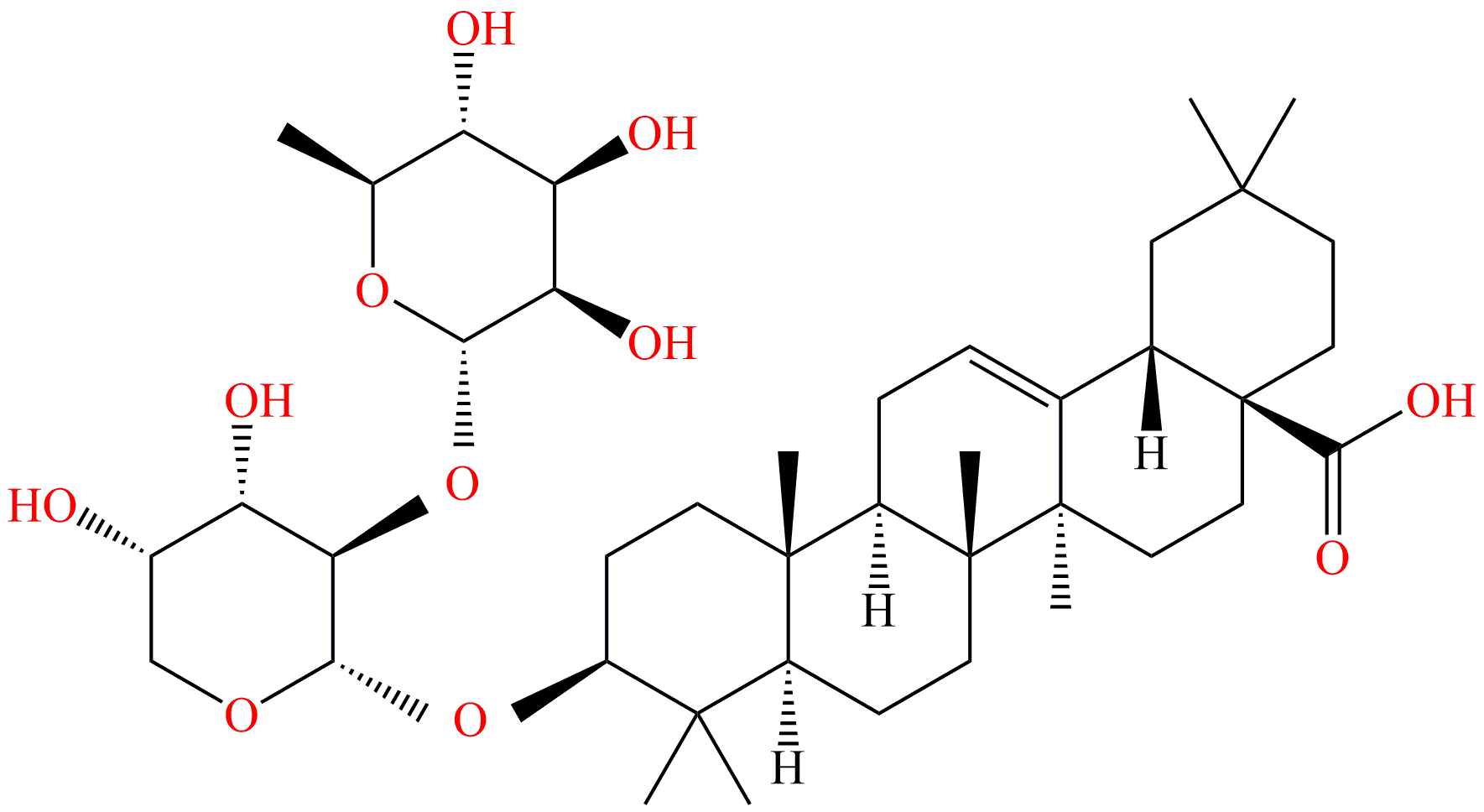


**Suppl. Figure** **1** The chemical structure of Eleutheroside K

Supplement: Supplementary file 1 — Supplementary file1 (DOCX 76 kb) [file 284_2021_2631_MOESM1_ESM.docx]
