# Supplementary material for: Eleutheroside K Isolated from Acanthopanax henryi (Oliv.) Harms Inhibits the Expression of Virulence-Related Exoproteins in Methicillin-Resistant Staphylococcus aureus
Source: Curr Microbiol. 2021 Sep 23;78(11):3980–8. doi: 10.1007/s00284-021-02631-5 (PMC8486718; doi:10.1007/s00284-021-02631-5)
Supplement: Supplementary file 2 — Supplementary file2 (DOCX 1375 kb) [file 284_2021_2631_MOESM2_ESM.docx]

1.


2.


3.


4.



**Suppl. Figure 2** The original, uncropped and minimally adjusted images of the gels and blots presented on Fig. 3. A, α-hemolysin (HLA); B, staphylococcal enterotoxin A (SEA); C, staphylococcal enterotoxin B (SEB), D, glyceraldehyde 3-phosphate dehydrogenase (GAPDH)
