# Supplementary material for: Eleutheroside K Isolated from Acanthopanax henryi (Oliv.) Harms Inhibits the Expression of Virulence-Related Exoproteins in Methicillin-Resistant Staphylococcus aureus
Source: Curr Microbiol. 2021 Sep 23;78(11):3980–8. doi: 10.1007/s00284-021-02631-5 (PMC8486718; doi:10.1007/s00284-021-02631-5)
Supplement: Supplementary file 3 — Supplementary file3 (DOCX 12 kb) [file 284_2021_2631_MOESM3_ESM.docx]

**Suppl. Table 1** Primers used in quantitative real-time polymerase chain reaction analyses

| Primer | Sequence |
| --- | --- |
| *sea* | Forward: 5'-ATGGTGCTTATTATGGTTATC-3' |
|  | Reverse: 5'-CGTTTCCAAAGGTACTGTATT-3' |
| *seb* | F: 5'-TGTTCGGGTATTTGAAGATGG-3' |
|  | R: 5'-CGTTTCATAAGGCGAGTTGTT-3' |
| *hla* | F: 5'-TTGGTGCAAATGTTTC -3' |
|  | R: 5'-TCACTTTCCAGCCTACT -3' |
| *agrA* | F: 5'-TGATAATCCTTATGAGGTGCTT-3' |
|  | R: 5'-CACTGTGACTCGTAACGAAAA-3' |
| *16S RNA* | F: 5'-GCTGCCCTTTGTATTGTC-3' |
|  | R: 5'-AGATGTTGGGTTAAGTCCC-3' |
